# The amphipathic helices of Arfrp1 and Arl14 are sufficient to determine subcellular localizations

**DOI:** 10.1074/jbc.RA120.014999

**Published:** 2021-01-13

**Authors:** Feng Yang, Tiantian Li, Ziqing Peng, Yang Liu, Yusong Guo

**Affiliations:** 1 Division of Life Science, Hong Kong University of Science and Technology, Hong Kong, China; 2 Hong Kong University of Science and Technology Shenzhen Research Institute, Shenzhen, China

**Keywords:** ARF, GTPase, Golgi, endosome, sorting, ADP ribosylation factor (ARF)

## Abstract

The subcellular localization of Arf family proteins is generally thought to be determined by their corresponding guanine nucleotide exchange factors. By promoting GTP binding, guanine nucleotide exchange factors induce conformational changes of Arf proteins exposing their N-terminal amphipathic helices, which then insert into the membranes to stabilize the membrane association process. Here, we found that the N-terminal amphipathic motifs of the Golgi-localized Arf family protein, Arfrp1, and the endosome- and plasma membrane–localized Arf family protein, Arl14, play critical roles in spatial determination. Exchanging the amphipathic helix motifs between these two Arf proteins causes the switch of their localizations. Moreover, the amphipathic helices of Arfrp1 and Arl14 are sufficient for cytosolic proteins to be localized into a specific cellular compartment. The spatial determination mediated by the Arfrp1 helix requires its binding partner Sys1. In addition, the residues that are required for the acetylation of the Arfrp1 helix and the myristoylation of the Arl14 helix are important for the specific subcellular localization. Interestingly, Arfrp1 and Arl14 are recruited to their specific cellular compartments independent of GTP binding. Our results demonstrate that the amphipathic motifs of Arfrp1 and Arl14 are sufficient for determining specific subcellular localizations in a GTP-independent manner, suggesting that the membrane association and activation of some Arf proteins are uncoupled.

The small GTPases of the ADP-ribosylation factor (Arf) family are key regulators of protein-sorting processes in the secretory and endocytic transport pathways (1, 2). Arf family proteins mainly function to mediate recruitment of cytosolic effectors to specific membrane compartments. This process brings their effectors in close proximity to membranes, facilitating Arf effectors to perform cargo recognition, lipid modification, or other cellular functions (1, 2).

Arf family proteins cycle between a GDP-bound inactive state and a GTP-bound active state. They have similar structural organizations containing an N-terminal amphipathic helix motif and the switch domains. The switch domains of Arf proteins directly bind their corresponding guanidine nucleotide exchange factors (GEFs), thus enabling Arf proteins to bind GTP (3). Structural analysis reveals that the amphipathic motif is held in a hydrophobic pocket in the GDP-bound status (4). Upon GTP binding, Arf family proteins undergo conformational changes, eliminating the binding of the N-terminal amphipathic helix to the hydrophobic pocket, thereby releasing the N-terminal amphipathic helix out of the pocket (3, 5). After being exposed outside of the pocket, the amphipathic helix will then insert into the membranes to stabilize the membrane association of the Arfs. GTP binding also causes conformational changes of the switch domains to mediate membrane recruitment of their cytosolic effectors.

In mammalian cells, there are 5 Arf proteins and ∼20 Arf-like proteins (1, 2). Many of the Arf family proteins and their corresponding Arf GEFs are localized on specific cellular compartments. An Arf family protein, Arfrp1, localizes to the *trans* Golgi network and regulates trafficking of various cargo proteins including tyrosine kinase 7 (PTK7), E-cadherin, Vangl2, and glucose transporters GLUT2 and GLUT4 (6, 7, 8, 9, 10, 11, 12). Arfrp1 also functions upstream of two other Arf family proteins, Arl1 and Arl5, to mediate membrane recruitment of tethering factors, and this process plays an important role in mediating tethering of retrograde carriers to the TGN (13). Arl14 is an Arf protein that regulates movement of MHC-II vesicles along the actin cytoskeleton in human dendritic cells, and PSD4 is implicated to function as a GEF to promote GTP binding of Arl14 (14).

It is generally conceived that membrane recruitment of Arf proteins is initiated by encountering GDP-bound Arf proteins with their specific GEFs. This process determines the specific localizations of Arf family proteins. In this study, we investigated roles of the amphipathic helix motifs of Arf proteins in determining their specific subcellular localizations. Interestingly, we found that the amphipathic helix motifs of Arfrp1 and Arl14 are sufficient to determine spatial localizations. The residues that are required for the acetylation modification on Arfrp1 helix and the myristoylation modification on Arl14 helix are important for the spatial determination. In addition, these Arf proteins can be recruited to the membranes independent of GTP. Our analysis provides a novel mechanism for membrane association of some Arf proteins, in which the amphipathic helix motif plays critical roles in determining specific subcellular localization in a GTP-independent manner. We hypothesize that this GTP-independent membrane recruitment process will increase the efficiency of Arf proteins to interact with their corresponding GEFs, thereby facilitating their activities to regulate membrane recruitment of downstream factors.

## Results

### The amphipathic helices of Arfrp1 and Arl14 are important for determining specific subcellular localizations

**Figure 1:**
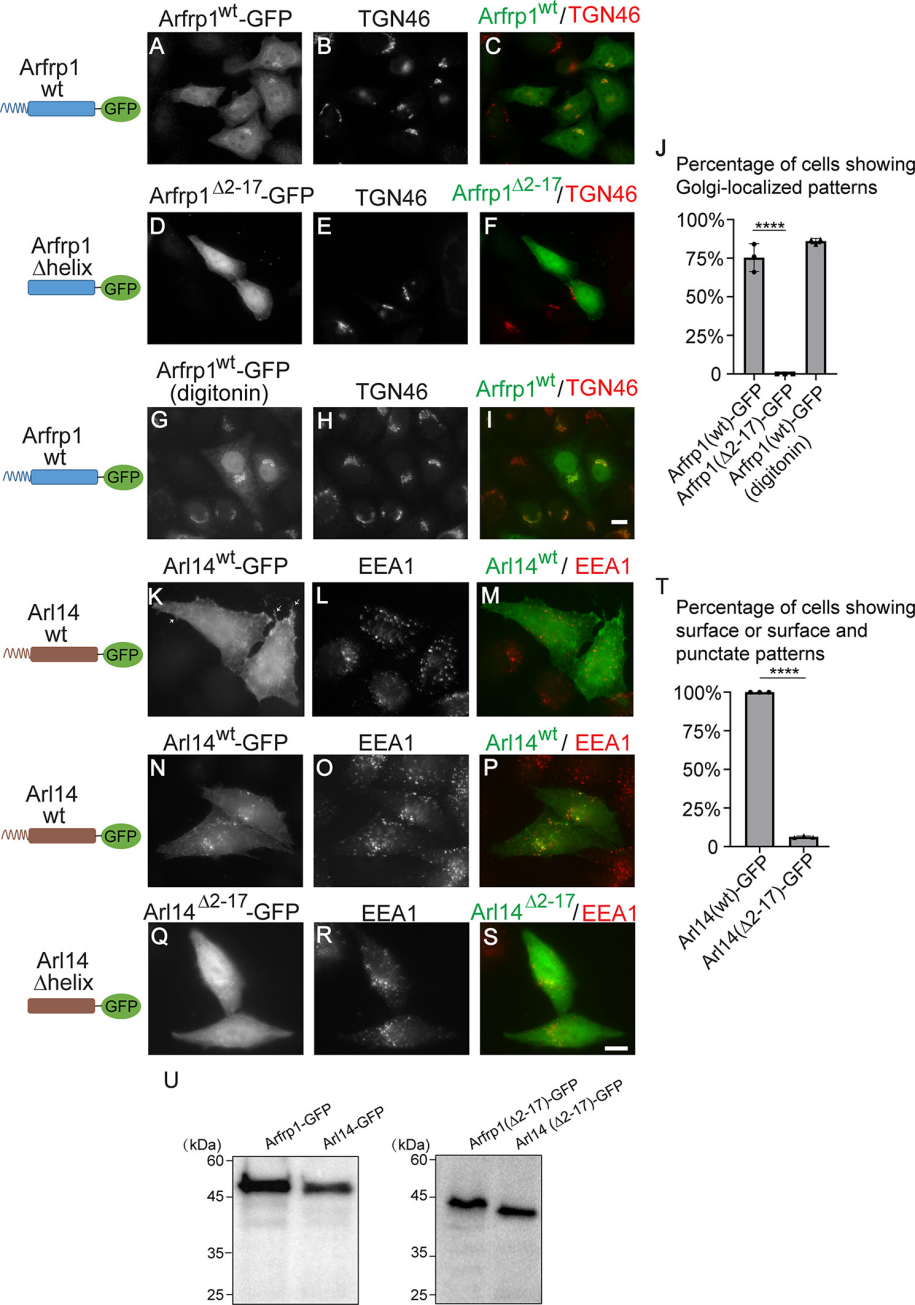
**The amphipathic helix motifs of Arfrp1 and Arl14 are important for their membrane associations.** HeLa cells were transiently transfected with plasmids encoding the indicated constructs (*A–I* and *K–S*). 10 h after transfection, the localizations of the indicated proteins were analyzed by immunofluorescence. *Scale bar*, 10 μm. The percentage of cells showing Golgi-localized patterns of the indicated constructs was quantified (*J*, *n* = 3, mean ± S.D., >100 cells counted in each experiment). The percentage of cells showing surface or surface and punctate patterns of the indicated constructs was quantified (*T*, *n* = 3, mean ± S.D., >100 cells counted in each experiment). ****, *p* < 0.0001. HeLa cells were transfected with plasmids encoding the indicated constructs. 10 h after transfection, GFP-tagged constructs in cell lysates were analyzed by immunoblot (*U*).

We selected two Arf proteins, Arfrp1 and Arl14, to study the functional roles of Arf amphipathic motifs. We used mammalian cells that overexpress GFP-tagged constructs for the localization analysis. GFP-tagged Arfrp1 (Arfrp1–GFP) was localized at the Golgi and cytoplasm in the majority of the expressing cells (Fig. 1, *A–C*; quantification is shown in Fig. 1*J*). Golgi-localized Arfrp1–GFP was more obviously detected if the cells were pretreated with digitonin to release the cytosolic pool of Arfrp1–GFP prior to fixation (Fig. 1, *G–I*; quantification is shown in Fig. 1*J*). GFP-tagged Arl14 showed a clear linear pattern on the cell boundary, indicating its localization at the plasma membrane in the majority of the expressing cells (Fig. 1, *K–M*). Arl14–GFP was also localized on the narrow extensions that extended toward the outside of the cell in some of the expressing cells (Fig. 1*K*, *arrows*). Some of the expressing cells also showed plasma membrane and intracellular punctate localization patterns (Fig. 1, *N–P*). The punctate localization pattern of Arl14–GFP partially colocalized with early endosomal marker EEA1 (Fig. 1, *N–P*).

To test whether the amphipathic helices of Arfrp1 and Arl14 contribute to the localization of these Arf proteins, we deleted the amphipathic helix motif of these two Arf proteins. We found that the GFP-tagged Arfrp1Δhelix (Arfrp1^Δ2–17^–GFP) had dissociated from the Golgi and showed a cytoplasmic and nucleus localization pattern in all of the expressing cells (Fig. 1, *D–F*; quantification is shown in Fig. 1*J*). Overexpression of Arfrp1^Δ2–17^–GFP caused dispersal of TGN46 from the juxtanuclear Golgi area (Fig. 1*E*). Arl14^Δ2–17^–GFP also lost its original localizations and showed cytoplasmic and nucleus localization patterns in the majority of the expressing cells (Fig. 1, *Q–S*; quantification is shown in Fig. 1*T*). Although GFP-tagged full-length Arfrp1 and Arl14 were stable, we detected degradation of GFP-tagged Arfrp1^Δ2–17^ and Arl14^Δ2–17^ on day 1 after transfection. Western blotting analysis showed no detectable degradation of the GFP-tagged constructs 10 h after transfection (Fig. 1*U*). Thus, all of the analysis in Fig. 1 was performed at this time point. The N-terminal amphipathic helix of Arf family proteins is often deleted to study the interaction between Arf proteins and the binding partners that interact with their switch domains. Although these interactions can take place, Arfrp1 and Arl14 constructs depleted of the N-terminal amphipathic helices lost their membrane associations, indicating that the amphipathic helix motifs are important for associations of Arfrp1 and Arl14 with specific cellular compartments.

### The amphipathic helices of Arfrp1 and Arl14 are sufficient to determine localization to specific cellular compartments

**Figure 2:**
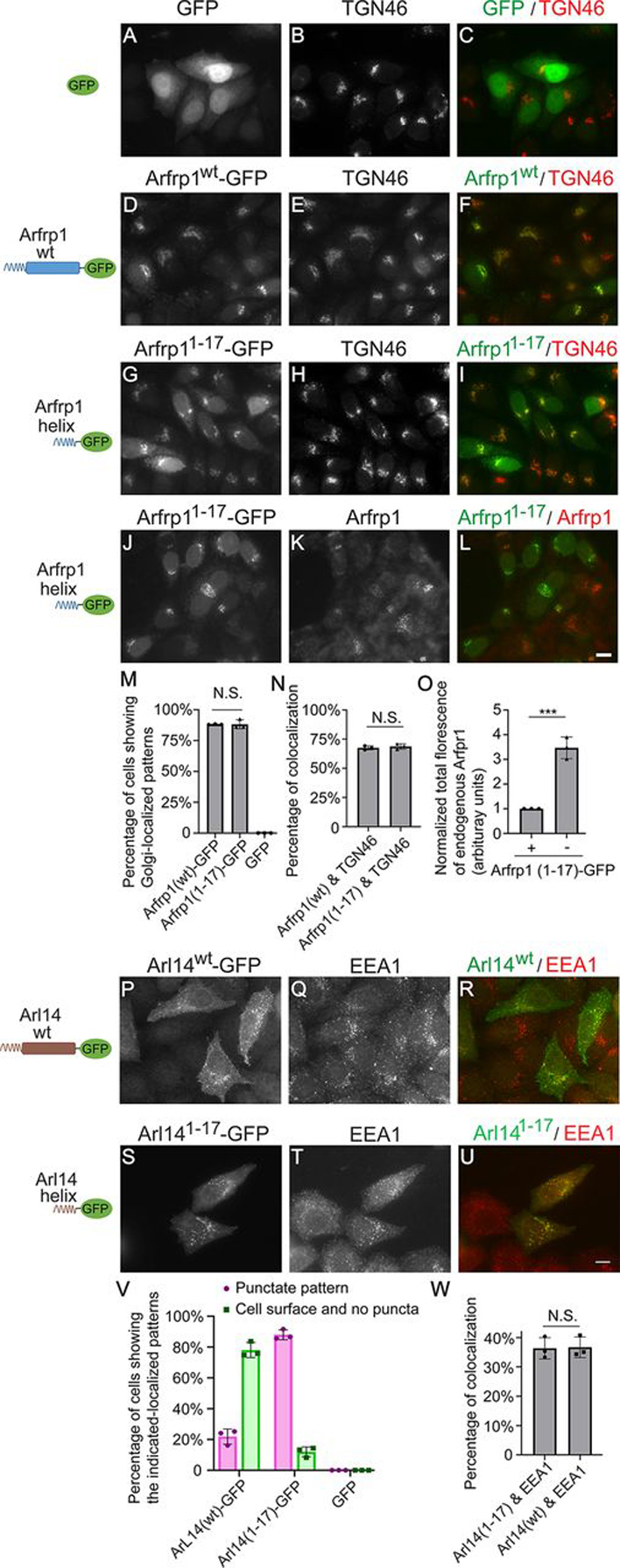
**The amphipathic helix motifs of Arfrp1 and Arl14 are sufficient to bring GFP to specific cellular compartments.** HeLa cells were transiently transfected with plasmids encoding the indicated constructs (*A–L* and *P–U*). Day 1 after transfection, the localizations of the indicated proteins were analyzed by immunofluorescence. *Scale bar*, 10 μm. The percentage of cells showing the indicated localization patterns of the GFP-tagged constructs was quantified (*M* and *V*, *n* = 3, mean ± S.D., >100 cells counted in each experiment). The percentage of TGN46 or EEA1 that was colocalized with the indicted constructs was quantified (*N* and *W*, *n* = 3, means ± S.D., >20 cells were quantified in each experiment). The total fluorescence of Arfrp1 in cells expressing or not expressing Arfrp1^1–17^–GFP was quantified (*O*, *n* = 3, means ± S.D., >20 cells were quantified in each experiment). The total fluorescence of Arfrp1 in each experimental group was normalized to that in cells expressing Arfrp1^1–17^–GFP. *N.S*., not significant; ***, *p* < 0.001.

Next we sought to test whether the amphipathic helix motifs of Arfrp1 and Arl14 are sufficient for determining localizations to specific cellular compartments. We generated a construct, Arfrp1^1–17^–GFP, in which the amphipathic helix motif of Arfrp1 was fused with GFP, and analyzed the localization of this fusion protein. When expressed alone, GFP was located at the cytoplasm and nucleus, and GFP showed no detectable localizations at the Golgi (Fig. 2, *A–C*; quantification is shown in Fig. 2*M*). Strikingly, we observed that Arfrp1^1–17^–GFP showed a similar localization pattern of GFP-tagged full-length Arfrp1 (Fig. 2, *D–I*). Quantification analysis indicates that Arfrp1^1–17^–GFP in >85% of the expressing cells showed a juxtanuclear Golgi-localized pattern, which was similar to that detected in cells expressing Arfrp1–GFP (Fig. 2*M*). The percentage of TGN46 that colocalized with Arfrp1^1–17^–GFP was similar to the percentage of TGN46 that colocalized with Arfrp1–GFP (Fig. 2*N*). A similar localization pattern was also detected when the amphipathic helix motif of Arfrp1 was fused to an IgG-binding ZZ domain and a FLAG tag (Arfrp1^1–17^–FLAG-ZZ; Fig. S1, G–I). Quantification analysis indicates that >90% of the expressing cells showed a Golgi-localized pattern of Arfrp1^1–17^–FLAG-ZZ, which was similar to that observed in cells expressing full-length Arfrp1–FLAG-ZZ (Fig. S1, D–F; quantification is shown in Fig. S1J). FLAG-ZZ, when expressed alone, was located at the nucleus and cytoplasm with no detectable Golgi-localized patterns (Fig. S1, A–C; quantification is shown in Fig. S1J). These results indicate that the amphipathic helix motif of Arfrp1 was sufficient to bring cytosolic proteins to the Golgi. Interestingly, expression of Arfrp1^1–17^–GFP caused dissociation of endogenous Arfrp1 from Golgi membranes (Fig. 2, *J–L*). Quantification analysis indicates that the total fluorescence of endogenous Arfrp1 in cells overexpressing Arfrp1^1–17^–GFP was significantly lower than that detected in cells not expressing Arfrp1^1–17^–GFP (Fig. 2*O*), suggesting that the amphipathic helix of Arfrp1 competes with endogenous Arfrp1 to be associated with Golgi membranes.

We then fused the amphipathic helix motif of Arl14 with GFP and analyzed the localization of the fusion protein (Arl14^1–17^–GFP). Arl14^1–17^–GFP showed punctate localization patterns in >80% of the expressing cells (Fig. 2, *S–U*; quantification is shown in Fig. 2*V*). We found that GFP-tagged full-length Arl14 showed punctate or punctate plus surface localization patterns in ∼20% of the expressing cells (Figs. 1, *N–P*, and Fig. 2, *P–R*; quantification is shown in Fig. 2*V*). In contrast, full-length Arl14–GFP in the majority (∼80%) of the expressing cells showed a cell surface–localized pattern with no detectable punctate structures (Fig. 1, *K–M*; quantification is shown in Fig. 2*V*). GFP, when expressed alone, did not show detectable surface or punctate localization patterns (Fig. 2*A*; quantification is shown in Fig. 2*V*). The punctate pattern of both Arl14^1–17^–GFP and Arl14–GFP partially colocalized with the early endosomal marker, EEA1 (Fig. 2, *P–U*). Quantification analysis indicates that the percentage of EEA1 that was colocalized with the punctate structures labeled by GFP-tagged full-length Arl14 was similar to that colocalized with the GFP-tagged amphipathic motif of Arl14 (Fig. 2*W*). We then generated constructs in which the full length or the N-terminal amphipathic helix of Arl14 was fused with the IgG-binding ZZ domain and a FLAG tag (Arl14–FLAG-ZZ or Arl14^1–17^–FLAG-ZZ). Both Arl14–FLAG-ZZ and Arl14^1–17^–FLAG-ZZ in almost all of the expressing cells showed a surface-localized pattern with no detectable punctate patterns (Fig. S1, K–P; quantification is shown in Fig. S1Q). FLAG-ZZ, when expressed alone, showed no detectable surface-localized patterns (Fig. S1A; quantification is shown in Fig. S1Q). We hypothesize that the configuration of the amphipathic helix of Arl14 may play an important role in regulating its subcellular localization, which is discussed under “Discussion.”

We also fused the amphipathic helix motifs of two other Golgi-localized Arf proteins, Arf1 and Arl1 with GFP, and analyzed the localization of the fusion proteins. GFP-tagged WT Arf1 and Arl1 were located at the juxtanuclear Golgi area in ∼80% of expressing cells (Fig. S2, A–C and G–I; quantification is shown in Fig. S2M). GFP-tagged Arf1 amphipathic helix and Arl1 amphipathic helix did not show a Golgi-localized pattern in any of the expressing cells (Fig. S2, D–F and J–L; quantification is shown in Fig. S2M), suggesting that the amphipathic helix motifs from these two Arfs are not sufficient for the determination of specific subcellular localizations.

### The residues that are required for the acetylation and myristoylation modifications on Arfrp1 helix and Arl14 helix are important for the spatial determination mediated by Arfrp1 helix and Arl14 helix

**Figure 3:**
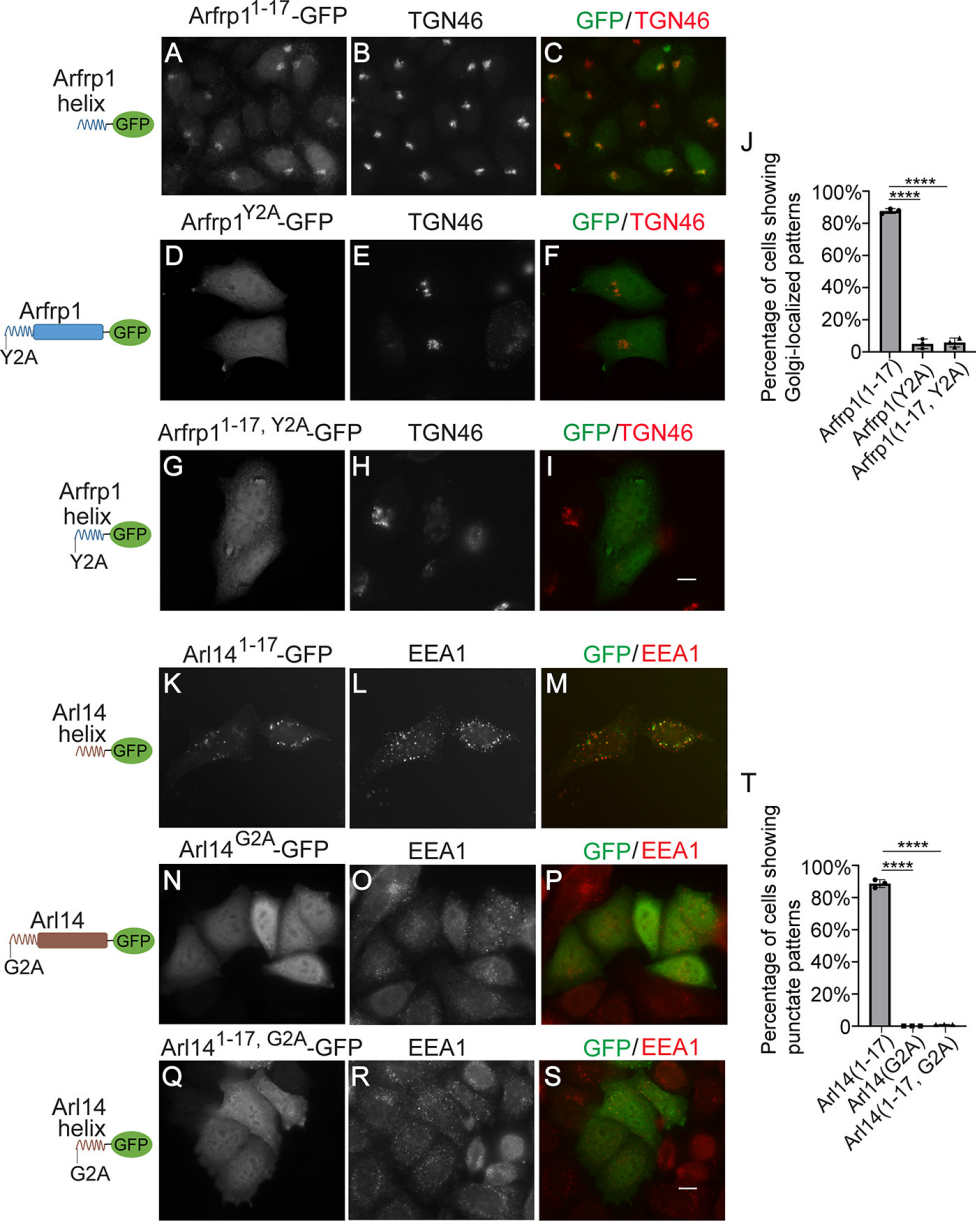
**The residues that are required for the acetylation and myristoylation modifications on Arfrp1 helix and Arl14 helix are important for the spatial determination mediated by Arfrp1 helix and Arl14 helix.** HeLa cells were transiently transfected with the indicated constructs (*A–I* and *K–S*). Day 1 after transfection, the localizations of the indicated proteins were analyzed by immunofluorescence. *Scale bar*, 10 μm. The percentage of cells showing Golgi or punctate localization patterns of the indicated constructs was quantified (*J* and *T*, *n* = 3, means ± S.D., >100 cells counted in each experiment). ****, *p* < 0.0001.

Our analysis indicates that the amphipathic helix motifs of Arfrp1 and Arl14 can interact with specific organelle membranes. Arfrp1 amphipathic helix and Arl14 amphipathic helix are acetylated and myristoylated, respectively (2). To test whether the acetylation and myristoylation modifications are required for their localizations, we generated mutant forms of Arfrp1 and Arl14 (Arfrp1^Y2A^–GFP and Arl14^G2A^–GFP) to block these modification processes and tested their localizations in HeLa cells. We found that both of these mutant constructs showed cytoplasmic localization patterns (Fig. 3, *D–F* and *N–P*). Similarly, mutating these residues in the GFP-tagged Arfrp1 N-terminal amphipathic helix and GFP-tagged Arl14 N-terminal amphipathic helix constructs caused dispersal of these two constructs from membranes (Fig. 3, *G–I* and *Q–S*). GFP-tagged Arfrp1 amphipathic helix and Arl14 amphipathic helix showed a Golgi- or endosome-localized pattern in >80% of the expressing cells (Fig. 3, *A–C* and *K–M*; quantification is shown in Fig. 3, *J* and *T*). In contrast, the acetylation- and myristoylation-defective constructs lost their localizations on specific organelles in the majority of expressing cells (Fig. 3, *J* and *T*). These analyses provide evidence suggesting that the spatial determination mediated by Arfrp1 helix and Arl14 helix requires the acetylation and myristoylation modifications on Arfrp1 helix and Arl14 helix, respectively.

### Sys1 is critical for recruiting Arfrp1 amphipathic helix to the Golgi

**Figure 4:**
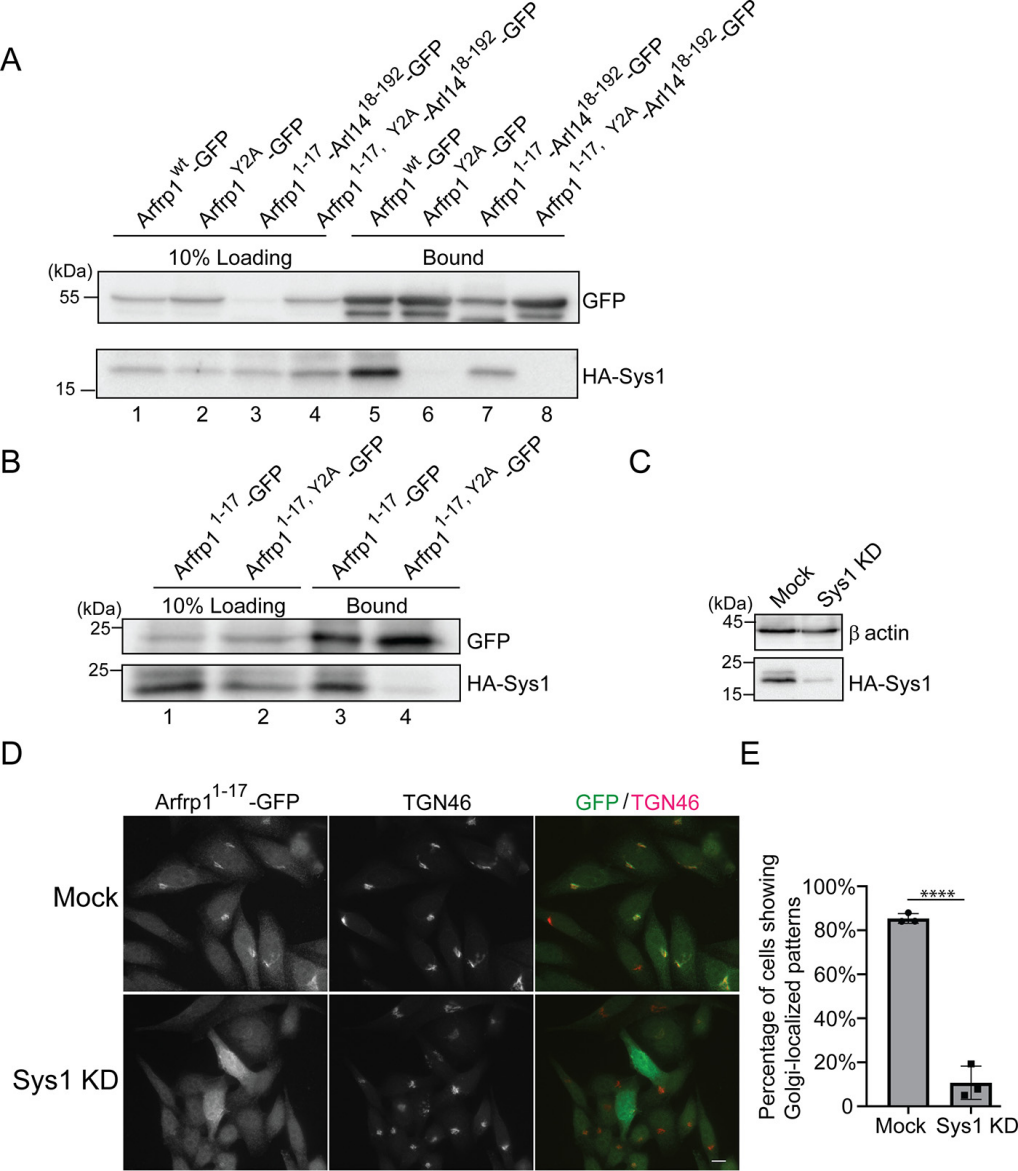
**Sys1 interacts with the amphipathic motif of Arfrp1 and is critical for recruiting the Arfrp1 amphipathic helix to the Golgi.** GFP trap beads were incubated with cell lysates from HeLa cells expressing the indicated constructs. After incubation, the bound proteins were analyzed by Western blotting using the indicated antibodies (*A* and *B*). HeLa cells were transfected with siRNA against Sys1. Day 3 after transfection, the cells were lysed and analyzed by Western blotting (*C*). HeLa cells were transfected with siRNA against Sys1. Day 2 after transfection, the cells were retransfected with Arfrp1^1–17^–GFP. 24 h after transfecting with DNA, the localizations of the indicated proteins were analyzed by immunofluorescence (*D*). *Scale bar*, 10 μm. The percentage of cells showing Golgi-localized patterns of Arfrp1^1–17^–GFP was quantified (*E*, *n* = 3, mean ± S.D., >100 cells counted in each experiment). ****, *p* < 0.0001. *KD*, knockdown.

Arfrp1 is acetylated on the amphipathic helix by the N-terminal acetyltransferase, NatC, and then the acetylated Arfrp1 interacts with the Golgi-localized Sys1 (15, 16). This interaction is important for recruitment of Arfrp1 to the Golgi membranes (15, 16). Moreover, the N-terminal helix of Arfrp1 is required for recruitment by Sys1 (15). Consistently, Arfrp1 coimmunoprecipitated with HA-tagged Sys1 and a Y2A mutation on Arfrp1 blocked the interaction (Fig. 4*A*, compare *lanes 5* and *6*). Interestingly, we found that the chimeric construct of Arfrp1 helix that was fused to Arl14Δhelix–GFP (Arfrp1^1–17^-Arl14^18-192^–GFP) or fused to GFP (Arfrp1^1–17^–GFP) also coimmunoprecipitated with HA-Sys1 (Fig. 4, *A*, *lane 7*, and *B*, *lane 3*), and Y2A mutations on Arfrp1 helix blocked this interaction (Fig. 4, *A*, *lane 8*, and *B*, *lane 4*). These results suggest that Sys1 interacted with Arfrp1 amphipathic helix. Knockdown of Sys1 caused dissociation of GFP-tagged Arfrp1 helix from the Golgi (Fig. 4, *C* and *D*; quantification is shown inFig. 4*E*). In contrast, Sys1 was not important for membrane recruitment of Arf1–GFP (Fig. S3A; quantification is shown in Fig. S3B). These results indicate that Sys1 is critical for recruiting the Arfrp1 amphipathic helix to the Golgi.

### Exchanging the amphipathic helix motifs between Arfrp1 and Arl14 causes the switch in their subcellular localizations

Our analyses indicate that the specific localizations of Arfrp1 and Arl14 can be determined by their N-terminal amphipathic helix motifs. Arf proteins contain switch domains that can directly interact with GEFs, and this interaction contributes to the localizations of Arf proteins (1). Thus, both the N-terminal amphipathic helix and the Arf/ArfGEF interaction contribute to the localization of Arl14 and Arfrp1. However, does the amphipathic motif of Arfrp1 and Arl14 play a dominant role in determining the localization of Arf proteins?

**Figure 5:**
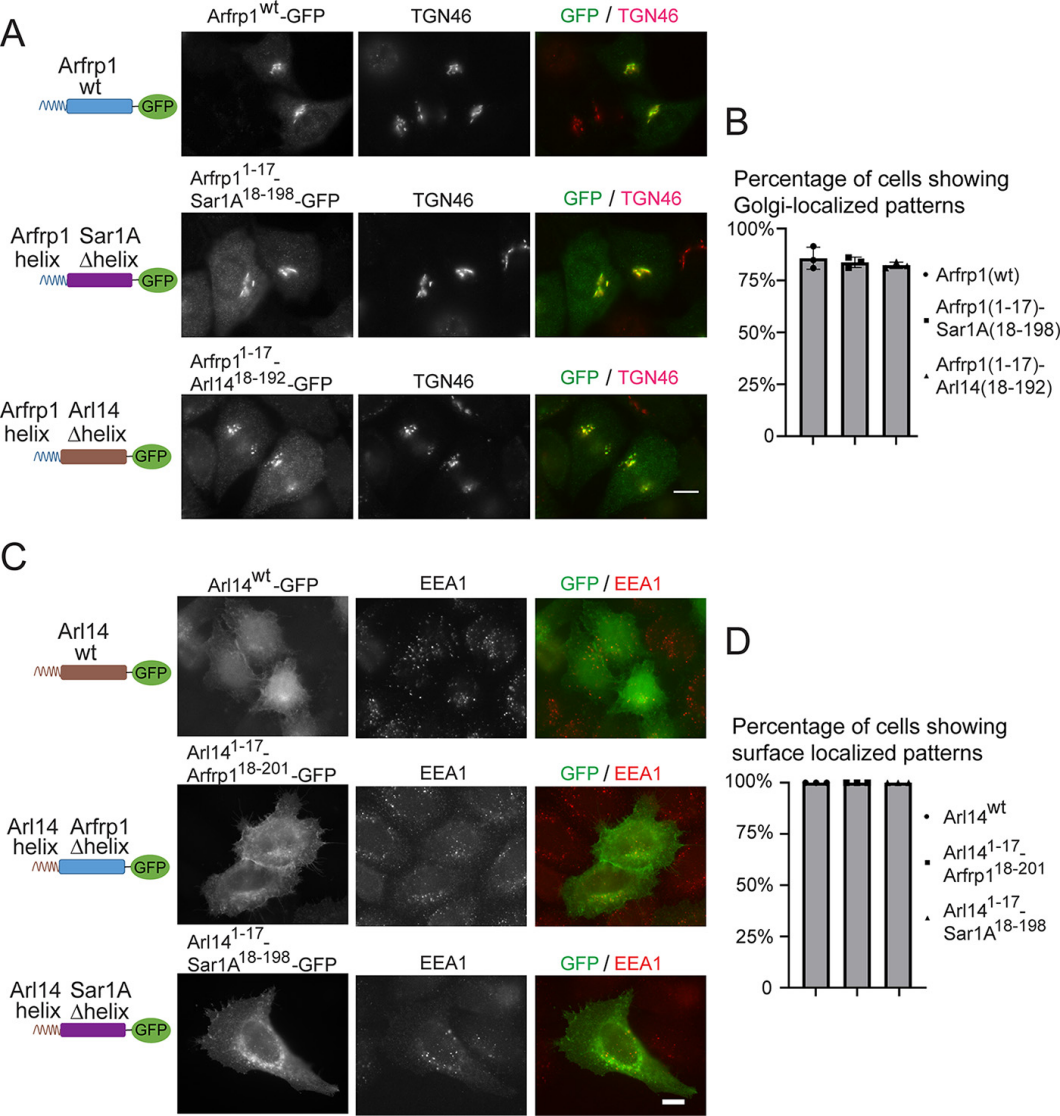
**Switching the amphipathic helix motifs among Arfrp1 and Arl14 causes changes of their localizations.** HeLa cells were transiently transfected with plasmids encoding the indicated constructs (*A* and *C*). Day 1 after transfection, the localizations of the indicated proteins were analyzed by immunofluorescence. *Scale bar*, 10 μm. The percentage of cells showing Golgi- or surface-localized patterns of the indicated constructs were quantified (*B* and *D*, *n* = 3, means ± S.D., >100 cells counted in each experiment).

To test this, we exchanged the amphipathic helix motifs between Arfrp1 and Arl14. We also replaced the amphipathic helix motif of an ER-located Arf family protein, Sar1A, with the amphipathic helix motif of Arfrp1 or Arl14. Interestingly, when Arfrp1 helix was fused to Arl14Δhelix–GFP and Sar1AΔhelix–GFP (Arfrp1^1–17^–Arl14^18–192^–GFP and Arfrp1^1–17^–Sar1A^18–198^–GFP), these two chimeric constructs showed a Golgi-localized pattern in >75% of expressing cells, which is similar to the localization pattern of Arfrp1–GFP (Fig. 5, *A* and *B*).

Arl14^1–17^–Arfrp1^18–201^–GFP and Arl14^1–17^–Sar1A^18–198^–GFP were not localized at the Golgi but instead were partially localized to the plasma membrane in all of the expressing cells (Fig. 5, *C* and *D*). Similar to Arl14^1–17^–FLAG-ZZ (Fig. S1, N–P), these chimeric constructs did not show obvious endosomal localization patterns. These results indicate that switching the amphipathic helix motifs between Arfrp1 and Arl14 causes changes in their localizations to specific cellular compartments.

### Arfrp1 and Arl14 are recruited to their specific cellular compartment independent of GTP binding

**Figure 6:**
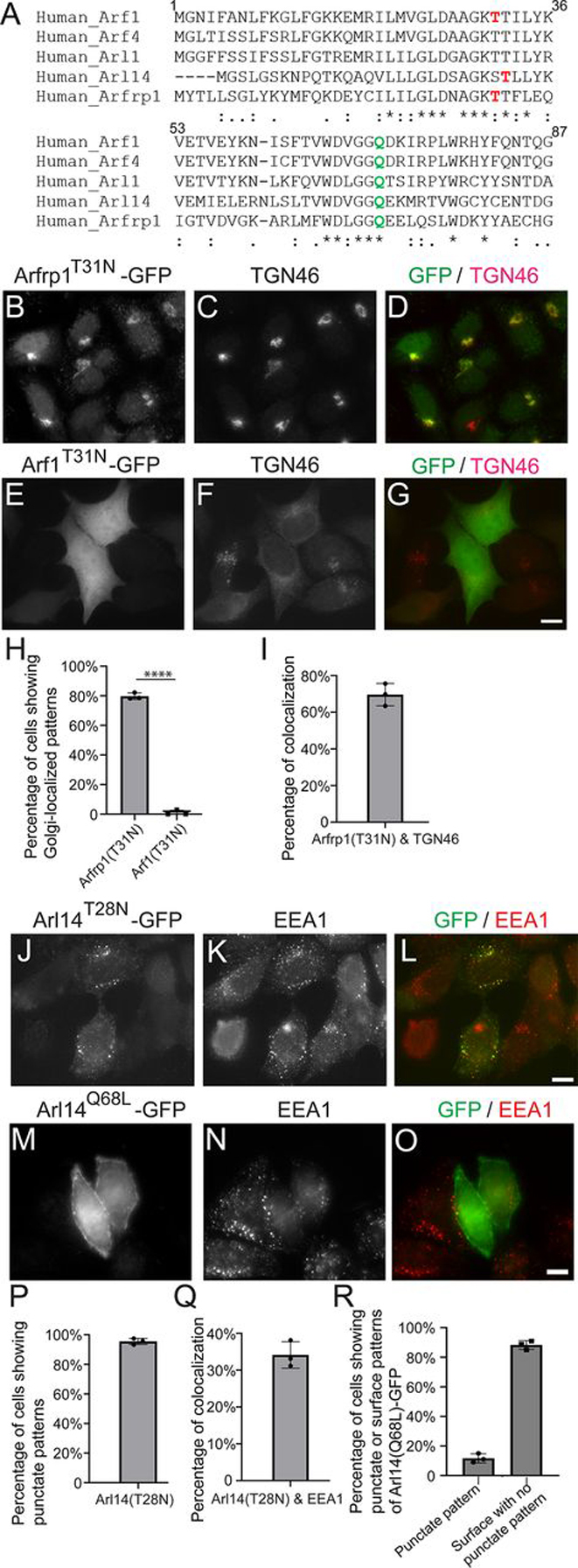
**The GDP-locked form of Arfrp1 and Arl14 can be recruited to their specific cellular compartments.** Sequence alignment of human Arf1, Arf4, Arl1, Arl14, and Arfrp1 is shown in *A*. HeLa cells were transiently transfected with plasmids encoding the indicated constructs (*B–G* and *J–O*). Day 1 after transfection, the localizations of the indicated proteins were analyzed by immunofluorescence. *Scale bar*, 10 μm. The percentage of cells showing the indicated subcellular colocalizations of the indicated constructs was quantified (*H*, *P*, and *R*, *n* = 3, means ± S.D., >100 cells counted in each experiment). ****, *p* < 0.0001. The percentage of TGN46 or EEA1 that was colocalized with the indicated constructs was quantified (*I* and *Q*, *n* = 3, means ± S.D., >20 cells quantified in each experiment).

The localizations of Arf family proteins are generally thought to be determined by Arf GEFs. We observed that exchanging the amphipathic helix motifs of other Arf proteins with that in Arfrp1 and Arl14 caused changes in the localization patterns of other Arf proteins (Fig. 5). This observation indicates that the amphipathic motifs of Arfrp1 and Arl14 can override the interaction between Arf and ArfGEF in determining the localization of Arf proteins. Next, we sought to test whether Arfrp1 and Arl14 are recruited to their specific cellular compartment independent of GTP binding. Threonine-to-asparagine mutations in the nucleotide-binding pocket inhibit GTP binding of many Arf family proteins (1). We generated GTP binding–deficient mutant versions of Arf1, Arl14, and Arfrp1 in which the threonine residue in the nucleotide-binding pocket (Fig. 6*A*, highlighted in *red*) was replaced by asparagine. Interestingly, we found that the GTP binding–deficient mutant versions of Arl14 and Arfrp1 still localized to the endosomes and the Golgi, respectively (Fig. 6, *B–D* and *J–L*; quantification is shown in Fig. 6, *H* and *P*). The percentage of TGN46 that was colocalized with Arfrp1^T31N^–GFP was similar to that colocalized with Arfrp1–GFP (Figs. 6*I* and 2*N*). The percentage of EEA1 that was colocalized with Arl14^T28N^–GFP was similar to that colocalized with the punctate structures of full-length Arl14–GFP (Fig. 6*Q* and 2*W*). In contrast, the GTP binding–deficient mutant forms of Arf1 had dissociated from the Golgi membranes (Fig. 6, *E–G*; quantification is shown in Fig. 6*H*). These results indicate that the GDP-locked form of Arfrp1 and Arl14 is recruited to the Golgi or endosomal membranes respectively.

We found that the majority of WT Arl14–GFP showed a surface-localized pattern with no detectable punctate structures (Fig. 2*V*), whereas the majority of the GDP-locked form, Arl14^T28N^–GFP, showed a punctate endosomal localization pattern (Fig. 6, *J–L* and *P*). Replacing the glutamine residue with leucine at the position 71 in human Arf1 reduces its intrinsic GTPase activity, causing Arf1 to be constitutively GTP-bound (17). Sequence alignment indicates that the Gln^71^ residue in human Arf1 is conserved in other human Arf family members (Fig. 6*A*, highlighted in *green*). We then generated a mutant version of Arl14–GFP in which the glutamine residue at the corresponding position was mutated to leucine (Arl14^Q68L^–GFP). Interestingly, Arl14^Q68L^–GFP showed a plasma membrane–localized pattern with no detectable endosomal punctate pattern in the majority of the expressing cells (Fig. 6, *M–O*; quantification is shown in Fig. 6*R*). These analyses indicate that the GTP- and GDP-locked form of Arl14 is preferentially located at the plasma membrane or endosomes, respectively.

**Figure 7:**
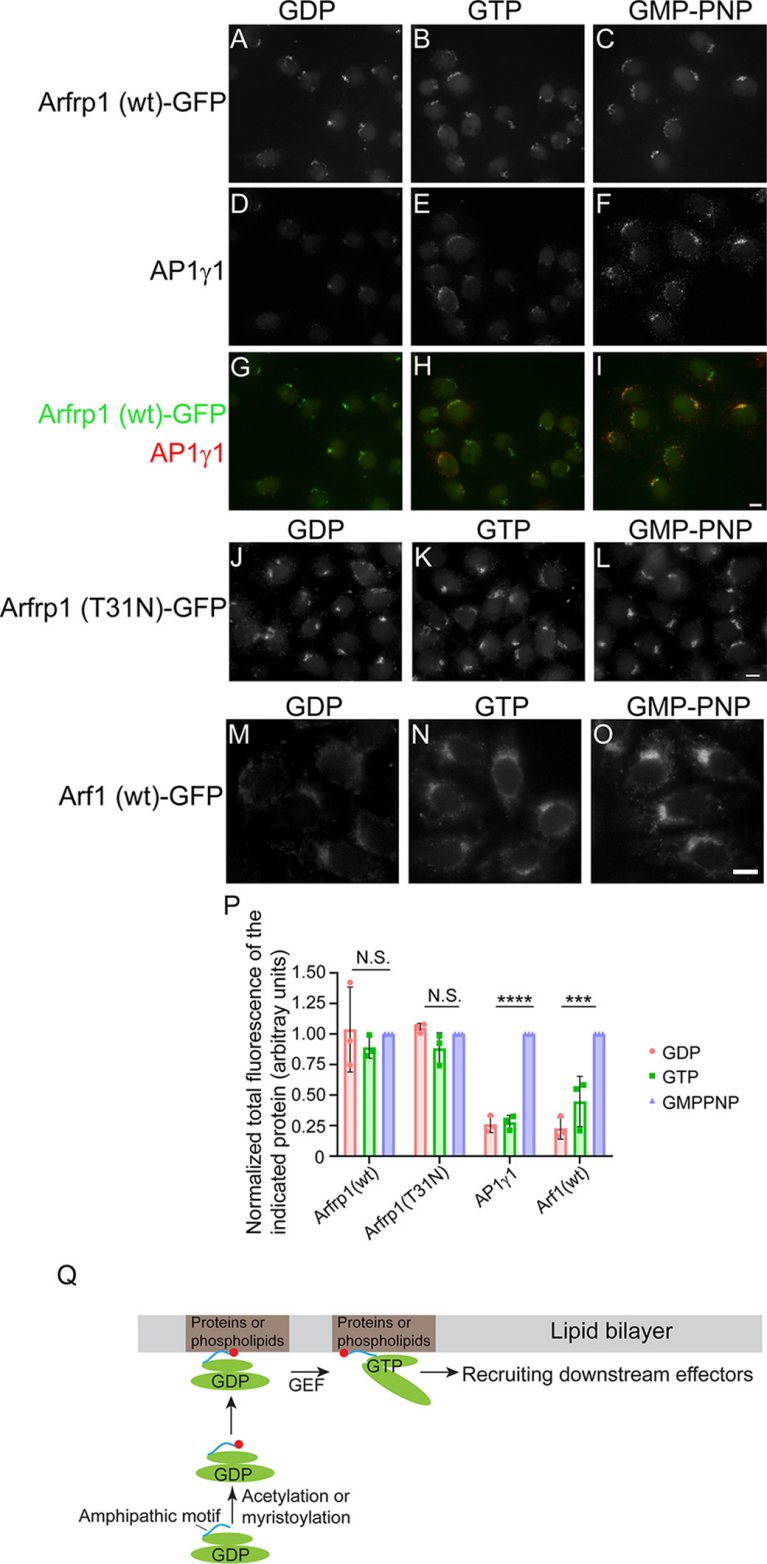
**Arfrp1 can be recruited to the Golgi independent of GTP binding.** HeLa cells were permeabilized by digitonin and incubated with the indicated reagents, and cytosol was prepared from HEK293T cells expressing Arfrp1^WT^–GFP (*A–C*), Arfrp1^T31N^–GFP (*J–L*), and Arf1–GFP (*M–O*). After incubation, the localization of the indicated proteins were analyzed by immunofluorescence. *Scale bar*, 10 μm. The total fluorescence of the indicated proteins were quantified in the experiment performed in the presence of the indicated nucleotides (*P*, *n* = 3, means ± S.D., >20 cells quantified in each experiment). The total fluorescence of each protein was normalized to that in the experiment performed in the presence of GMP-PNP in each experimental group. ***, *p* < 0.001; ****, *p* < 0.0001; *N.S.*, not significant. A proposed model of the membrane recruitment of Arfrp1 and Arl14 is shown in *Q*.

We then used a permeabilized cell assay to test whether membrane recruitment of these Arf proteins depends on GTP. HeLa cells were permeabilized by digitonin, salt-washed, and incubated with cytosol prepared from HEK293T cells expressing the GFP-tagged Arf proteins in the presence of GDP, GTP, or the nonhydrolyzable analog of GTP, GMP-PNP. Arl14–GFP cannot be recruited to the endosomal membranes in the presence of either GDP or GMP-PNP in this *in vitro* assay presumably because cholesterol in the plasma membrane and endosomes was disrupted. AP1γ1, GFP-tagged Arfrp1, Arfrp1^T31N^, and Arf1 were recruited to the juxtanuclear Golgi area in the presence of GMP-PNP (Fig. 7, *C*, *F*, *I*, *L*, and *O*). Because the exogenously added proteins were evenly distributed in all of the semi-intact cells in this assay, the staining intensities of these constructs at the Golgi area were relatively uniform. Interestingly, WT and the GTP binding–deficient mutant forms of Arfrp1 can still be recruited to the Golgi in the presence of GDP (Fig. 7, *A* and *J*). In contrast, Arf1 and AP1γ1 cannot be efficiently recruited to the Golgi membranes in the presence of GDP (Fig. 7, *D* and *M*). We then quantified the total fluorescence of the GFP-tagged Arf proteins or AP1γ1 that were recruited to the semi-intact cells in the presence of GDP, GTP, or GMPPNP. The quantification analyses indicate that GMP-PNP significantly enhanced the membrane recruitment of AP1γ1 and Arf1–GFP to the semi-intact cells (Fig. 7*P*). In contrast, the levels of Arfrp1–GFP and Arfrp1^T31N^–GFP that were recruited to the semi-intact cells were similar to that detected in presence of GDP or GMP-PNP (Fig. 7*P*). These results indicate that Arfrp1 is recruited to the membranes independent of GTP binding.

## Discussion

Arf family proteins are generally conceived to have an intrinsic property that their GTP binding and membrane association are tightly coupled (1, 2). In the GDP-bound status, the N-terminal amphipathic helix is buried to prevent its association with membranes. GTP-binding catalyzed by Arf-GEFs causes subsequent conformational changes exposing the amphipathic motif to bind lipids. This process is considered to be the major driving force that recruits Arf proteins to their specific cellular compartments. Here we found that associations of some Arf proteins, Arfrp1 and Arl14, with membranes occur in the absence of GTP binding. Similar to our observation, the GDP-locked form of the class II Arf protein, Arf4^T31N^, has been shown to be selectively associated with the ER-to-Golgi intermediate compartment (ERGIC) (18, 19). These observations suggest that GTP binding and membrane association of some Arf proteins are uncoupled. Interestingly, the amphipathic helices of Arfrp1 and Arl14 can bring GFP and the IgG-binding ZZ domain to specific cellular compartments, suggesting that they are sufficient for the spatial determination. Moreover, we found that switching the amphipathic helix of Arl14 and Arfrp1 causes the switch in their localization, suggesting that the amphipathic helix motifs can override the spatial localization determined by the binding between Arf-GEFs and Arf proteins. Our study indicates that these amphipathic motifs play critical roles in determining localizations to specific cellular compartments.

How can the amphipathic helices determine the specific localizations to cellular compartments? It has been reported that Golgi-localized Sys1 binds Arfrp1 to recruit Arfrp1 to Golgi membranes (15, 16). Binding of Sys1 to Arfrp1 depends on the amphipathic helix on Arfrp1 and mutating the acetylation modification blocks the interaction (15, 16). Here we found that Sys1 is important for recruitment of the Arfrp1 helix to the Golgi. The amphipathic helix of Arfrp1 is sufficient to bind Sys1, and the residue required for the acetylation modification is important for this binding. Thus, the spatial determination mediated by the Arfrp1 helix is mediated through an interaction with the Golgi localized protein, Sys1. It is currently unknown whether the amphipathic helices of Arl14 can interact with specific plasma membrane- or endosome-localized proteins for their spatial determinations. It is possible that the amphipathic motifs of Arl14 can also associate with specific phospholipids in the plasma membrane or the endosomes and thus can be recruited to specific compartments.

Arf proteins need to bind GTP to expose the hydrophobic surface of their amphipathic helix motifs to be tightly associated with membranes. Our results indicate that membrane association of Arfrp1 and Arl14 is independent of GTP binding. Why do some Arf proteins associate with membranes in the absence of GTP binding? Currently, the crystal structure of Arfrp1 and Arl14 is unknown. We hypothesize that it is the hydrophilic surface and not the hydrophobic surface of the amphipathic motif that attaches to its binding partner at the specific cellular compartment, thereby mediating membrane recruitment of these Arf proteins in the GDP-bound status. Arl14 contains a short amphipathic helix (2). It is possible that the hydrophobic surface of the amphipathic helix of Arl14 is not tightly associated with the hydrophobic pocket in the GDP-bound status. This enables the helix motif of Arl14 to remain flexible for association with lipid bilayers in the absence of GTP binding.

Interestingly, we found that the GDP- and GTP-locked forms of Arl14 are preferentially located at the endosome and plasma membrane, respectively. We propose that the configuration of the Arl14 amphipathic helix in the construct plays an important role in determining localizations at the plasma membrane or endosomes. If the configuration mimics the GDP-locked form of Arl14, it is preferentially located at the endosomes. If the configuration mimics the GTP-locked form of Arl14, it is preferentially located at the plasma membrane. WT Arf4 is shown to be located at the Golgi and ERGIC, whereas the GDP-locked form of Arf4, Arf4^T31N^, is preferentially located at the ERGIC (18), suggesting that a similar mechanism may also regulate the association of Arf4 to specific compartments. We observed that the N-terminal amphipathic helix motif of Arl14 can bring the GFP or IgG-binding ZZ domain to endosomes and plasma membrane, respectively. Based on this observation, we hypothesize that the configuration of the amphipathic helix of Arl14 in the GFP fusion construct mimics that in the GDP-bound form of Arl14, and the configuration of the amphipathic helix of Arl14 in the FLAG-ZZ fusion construct mimics that in the GTP-bound form of Arl14.

Altogether, we propose a model to explain the process of membrane recruitment of Arfrp1 and Arl14 (Fig. 7*Q*). First, the amphipathic helices of Arfrp1 and Arl14 will be acetylated or myristoylated. Subsequently, the modified amphipathic helix motifs of Arfrp1 and Arl14 will interact with the membrane-localized cellular factors, such as phospholipids or transmembrane proteins, to be recruited to the Golgi or endosomes, respectively. After being recruited to the membranes, the Arf proteins will meet with their corresponding GEF proteins to promote GTP binding and induce conformational changes of their switch domains. GTP-bound Arfrp1 then recruits downstream effector proteins to mediate vesicle formation or other cellular processes at the Golgi. In addition to promoting conformational changes of the switch domains of Arl14, GTP binding may cause a change of the configuration of the amphipathic helix of Arl14. This change induces its amphipathic helix to interact with the plasma membrane–localized cellular factors, such as cholesterol or cholesterol-modified proteins, to recruit Arl14 to the plasma membrane, where GTP-bound Arl14 will then recruit the downstream effector proteins. It is also possible that GTP-bound Arl14 interacts with specific plasma membrane–localized cellular factors on endosomes. This interaction in combination with the functions of other cellular machinery may mediate the sorting and surface delivery of these plasma membrane–localized cellular factors.

Association of Arf proteins with specific cellular compartments independent of GTP binding has several advantages. It increases the possibility of Arf proteins to meet with their specific GEF proteins for activation. GTP-independent association of Arf proteins with membranes will also allow Arf proteins, after GTP hydrolysis, to efficiently perform a further round of GTP binding and activation on membranes. The yeast homolog of Arfrp1, Arl3p, regulates membrane recruitment of Arl1p, which in turn recruits its effector GRIP domain protein Imh1p (20, 21). A similar cascade also exists in mammalian cells (11, 22). A recent study demonstrates that Sys1 and Arfrp1 regulate tethering of retrograde carriers to the TGN by functioning upstream of Arl1 and Arl5, which in turn regulates membrane recruitment of several golgins and GRAP, respectively, to regulate vesicle tethering (13). GTP-independent recruitment of Arfrp1 may increase the efficiency for Arfrp1 to initiate this sequential membrane recruitment process.

How are the Arf proteins extracted from membranes if they can be associated with membranes in a GTP-independent manner? For Rab proteins, GDP-bound Rab proteins are associated with a Rab GDP dissociation inhibitor to be extracted from membranes. We hypothesize that some unknown proteins can associate with the amphipathic motifs of Arfrp1 and Arl14 to extract them from membranes. In the tubular ER, a protein family named lunapark (Lnp) localizes at the three-way junctions. The N-terminal domain of Lnp contains an *N*-myristoylation site and coiled-coil domain 1 (CC1), which is predicted to form an amphipathic helix (23). The hydrophobic surface of the CC1 in the N-terminal domain of Lnp binds junction-enriched atlastins for proper localization, and this interaction compromises atlastin-mediated membrane fusion (23). It would be interesting to test whether the hydrophobic surface of the amphipathic helix of Arfrp1 and Arl14 interact with specific cellular factors and analyze the functional roles of these interactions.

Our study provides short motifs that are sufficient to bring cytosolic proteins to specific cellular compartments. These motifs will be potentially useful to target proteins to the plasma membrane, the Golgi, or the endosomes for functional studies.

## Materials and methods

### DNA constructs and antibodies

Plasmids encoding GFP-tagged WT or mutant versions of human Arf1, Arfrp1, Arf1, Arl1, Arl14, and Sar1A were cloned into pEGFP-N1 vector. Plasmids encoding the WT or mutant versions of human Arfrp1 and Arl14 fused to FLAG and an IgG-binding ZZ domain were generated by cloning into the modified pcDNA4/TO vector encoding FLAG tag and ZZ domain (kindly provided by the Zhong laboratory, Shanghai Jiaotong University, Shanghai, China). Human Sys1 with 3× HA tag at its C terminus was synthesized by BGI (Beijing, China) in pcDNA3.1 vector. The antibodies used in this study were as follows: mouse anti–GFP (Roche, catalog no. 11814460001, RRID:AB_390913); sheep anti-TGN46 (Bio-Rad catalog no. AHP500G, RRID:AB_323104); rabbit anti-Sys1 (Thermo Fisher Scientific catalog no. PA5-48935, RRID:AB_2634391); goat anti-EEA1 (Santa Cruz, catalog no. sc-6415; RRID:AB_2096822); mouse anti-Myc (Cell Signaling Technology catalog no. 2276, RRID:AB_331783); and rabbit anti-HA (Cell Signaling Technology catalog no. 3724, RRID:AB_1549585). The siRNA target sequence against Sys1 is TCTCCATGATGTCCTTCAT.

### Cell culture, transfection, and immunofluorescence

HeLa and HEK293T cells were cultured in Dulbecco's modified Eagle's medium containing 10% fetal bovine serum and 1% penicillin streptomycin mix (Invitrogen). DNA plasmids were transfected in HEK293T cells by polyethyleneimine. For HeLa cells, DNA plasmids were transfected using Lipofectamine® 2000 (Invitrogen).

To perform immunofluorescence experiments, cells cultured on 13-mm coverslips were fixed by 4% paraformaldehyde in PBS (137 mm NaCl, 2.7 mm KCl, 10 mm Na_2_HPO_4_, 1.8 mm KH_2_PO_4_, pH 7.4) at room temperature for 20 min. After fixation, the cells were then permeabilized and blocked by blocking buffer (1× PBS containing 0.2 m glycine, 2.5% FBS, and 0.1% Triton X-100) for 30 min at room temperature. Primary antibodies diluted in blocking buffer were added to coverslips and incubated for 1 h at room temperature. After incubation with primary antibodies, the cells were washed by PBS. Secondary antibodies diluted in blocking buffer were then added to the coverslips and incubated for 1 h at room temperature, followed by extensive washing by PBS. Finally, coverslips are mounted on glass slides with cells facing down using ProLong^TM^ Gold antifade mountant (Invitrogen, catalog no. P36930). The samples were observed under the Zeiss Axio Observer Z1 microscope (Carl Zeiss) equipped with an ORCA Flash 4.0 camera (Hamamatsu, Hamamatsu, Japan), and microscopic images were analyzed and processed using Fiji software.

### Quantification of the percentage of colocalization

The percentage of colocalization was quantified by calculating the percentage of overlapped pixels using Fiji based on a modified quantification method (24, 25). The quantification is performed using the following procedures: 1) a threshold was chosen manually based on the original gray scale images; 2) the divide function was used to equalize the average pixel intensity for the two thresholded images; 3) the measure function was used to determine the number of above-threshold pixels for each marker per cell; 4) the colocalization highlight function in the McMaster Biophotonics Facility (MBF) plugin collection was used to determine the number of above-threshold overlapped pixels with a fixed ratio of 0.6; and 5) in each cell, the number of overlapped pixels was divided by the number of above-threshold pixels of a marker to yield the percentage of a given maker's area in a cell that overlapped with the other marker.

### Permeabilized cell assays

The permeabilized cell assays were performed according to a previous report (26). HeLa cells grown on 13-mm glass coverslips were washed with cold KOAc buffer (110 mm KOAc, 2.5 mm MgOAc, 25 mm Hepes, pH 7.2). Then cells were permeabilized by 0.5 ml of 0.03 mg/ml digitonin in KOAc buffer for 6 min at room temperature. The permeabilized cells were washed with cold KOAc buffer. After 5 min of incubation on ice with 0.5 ml of cold 0.5 m KOAc buffer followed by three 0.5-ml washes in cold KOAc buffer to remove cytosolic proteins, the permeabilized cells were transferred to parafilm and incubated at 37 °C for 15 min in 50 μl of KOAc buffer containing needle lysed cytosol prepared from HEK293T cells expressing the GFP-tagged Arf constructs, 500 μm GDP/GTP/GMPPNP, and an ATP regeneration system (0.5 mm ATP, 0.5 mm UTP, 50 μm GTP, 5 mm creatine phosphate, 25 μg/ml creatine phosphokinase, 0.05 mm EGTA, and 0.5 mm MgCl_2_). The cells were then washed with cold KOAc buffer, fixed, and stained with specific antibodies.

## Data availability

All data are contained within the article and the supporting information.

10.13039/501100003802the Research Grants Council (RGC) of Hong Kong (16101116) to Yusong Guo
