# Supplementary material for: The amphipathic helices of Arfrp1 and Arl14 are sufficient to determine subcellular localizations
Source: J Biol Chem. 2021 Jan 13;295(49):16643–54. doi: 10.1074/jbc.RA120.014999 (PMC7864062; doi:10.1074/jbc.RA120.014999)
Supplement: Supplementary file 1 [file mmc1.pdf]

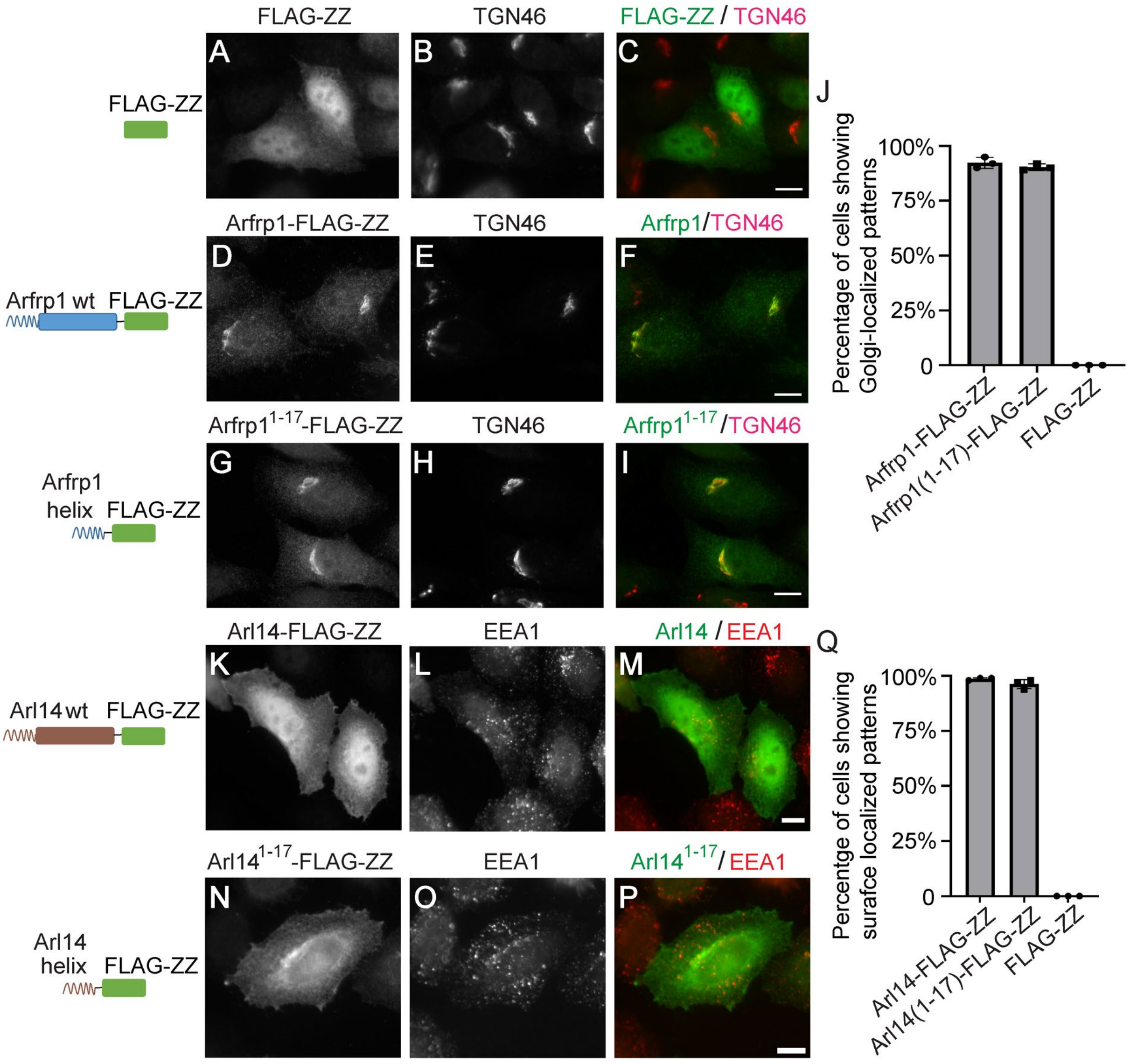

Figure S1. The amphipathic helices of Arfrp1 and Arl14 are sufficient to bring the IgG-binding ZZ domain to the Golgi and the plasma membrane respectively.

HeLa cells were transiently transfected with plasmids encoding the indicated constructs. Day 1 after transfection, the localizations of the indicated proteins were analyzed by immunofluorescence (A-I, K-P). Size Bar, 10 µm. The percentage of cells showing the indicated localization patterns of the indicated constructs was quantified (J and Q, n=3, mean ±S.D., over 100 cells quantified in each experiment).

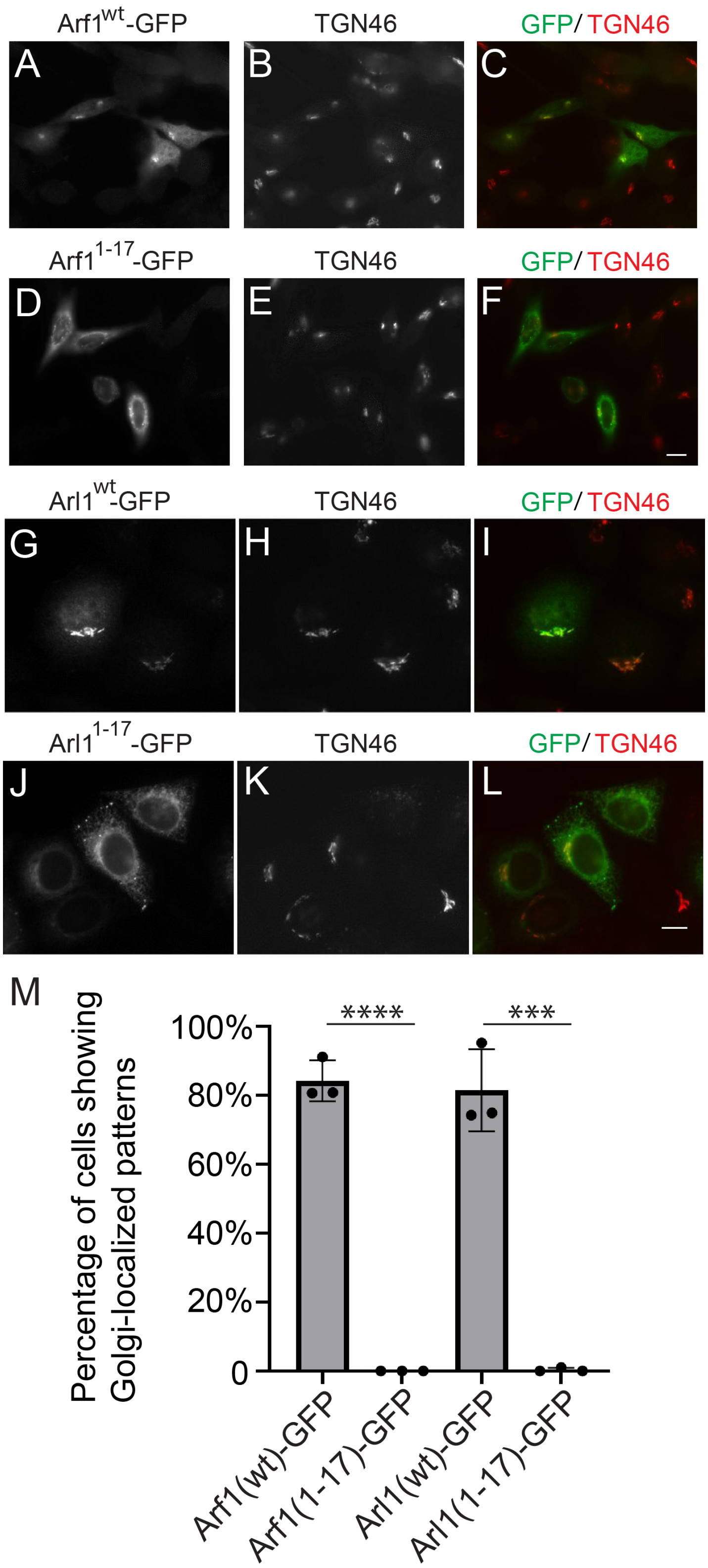

Figure S2. The amphipathic helices of Arf1 and Arl1 cannot bring GFP to the Golgi.
HeLa cells were transiently transfected with plasmids encoding the indicated constructs (A-L). Day 1 after transfection, the localizations of the indicated proteins were analyzed by immunofluorescence. Size Bar, 10μm. The percentage of cells showing juxtanuclear Golgi-localized pattern of the indicated protein was quantified (M, n=3, mean ±S.D., over 100 cells quantified in each experiment). ***, p<0.001; ****, p<0.0001.

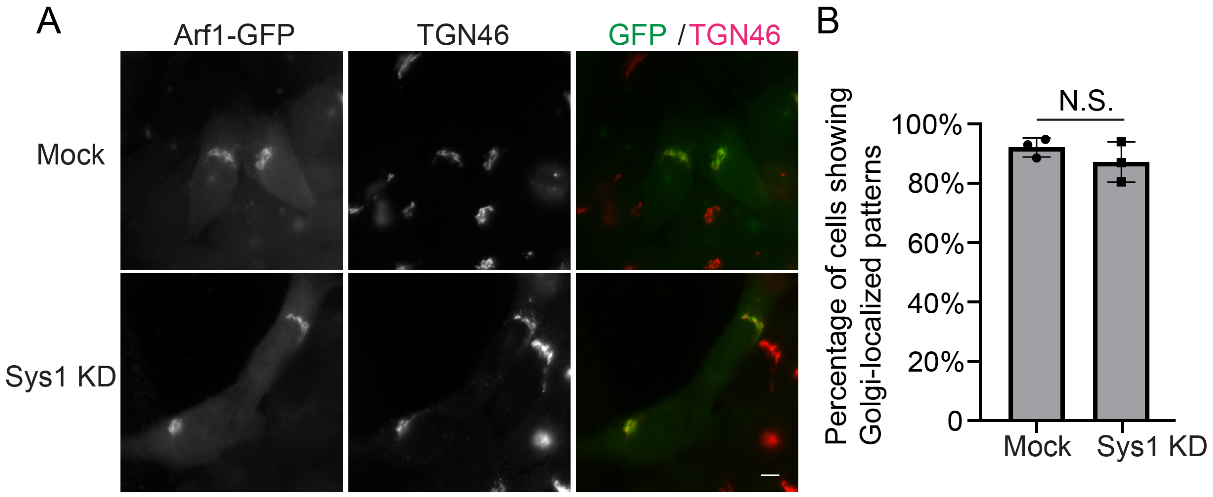

Figure S3. Knockdown of Sys1 does not cause defects in localizations of Arf1-GFP.
HeLa cells were transfected with siRNA against Sys1. Day 2 after transfection, cells were re-transfected with Arf1-GFP. 24 h after transfecting with DNA, the localizations of the indicated proteins were analyzed by immunofluorescence (A). Size Bar, 10 µm. The percentage of cells showing Golgi-localized patterns of Arf1-GFP in control and knockdown cells was quantified (B, n=3, mean ±S.D., over 100 cells quantified in each experiment). N.S., not significant.
